# Addressing Profound Disadvantages to Improve Indigenous Health and Reduce Hospitalisation: A Collaborative Community Program in Remote Northern Territory

**DOI:** 10.3390/ijerph16224306

**Published:** 2019-11-06

**Authors:** Simon Quilty, Lisa Wood, Sophie Scrimgeour, Geordan Shannon, Elisha Sherman, Bruce Lake, Richard Budd, Paul Lawton, Mary Moloney

**Affiliations:** 1 Department of Medicine, Alice Springs Hospital, Alice Springs 0870, Australia; 2 School of Population and Global Health, University of Western Australia, Perth 6009, Australia; 3 Royal Darwin Hospital, Darwin 0810, Australia; 4 Institute of Global Health, University College London, London WC1N 1EH, UK; 5 Wurli-Wurlinjang Aboriginal Health Service, Katherine 0850, Australia; 6 Katherine Hospital, Katherine 0850, Australia; 7 Menzies School of Health Research, Darwin 0810, Australia

**Keywords:** indigenous health, frequent attender, emergency department, homelessness, tropical environment

## Abstract

Background: Aboriginal people in rural and remote areas of the Northern Territory of Australia have suffered longstanding issues of homelessness and profound health and social inequities. The town and region of Katherine are particularly impacted by such inequities and have the highest rates of homelessness in Australia, composed almost entirely of Aboriginal people who represent 51% of the total population of 24,000 people. The region is serviced by a 60-bed hospital, and a small cohort of frequent attenders (FAs) represent 11% of the Emergency Department (ED) case load. The vast majority of FAs are Aboriginal and have very high burdens of social inequity and homelessness. FAs are a challenge to efficient and effective use of resources for most hospitals around the world, and investment in programs to address underlying social and chronic health issues contributing to frequent attendance have been demonstrated to be effective. Methods: These are the interim findings of a prospective cohort study using five sources of linked health and related data to evaluate a community-based case management pilot in a culturally competent framework to support frequent attenders to the Katherine Hospital ED. FAs were defined as people with six or more presentations in 12 preceding months. The intervention composed of a community-based case management program with a multi-agency service delivery addressing underlying vulnerabilities contributing to ED presentations. Results: Among this predominantly Aboriginal cohort (91%), there were high rates of homelessness (64%), food insecurity (60%) and alcohol misuse (64%), limited access to transport, and complex comorbidities (average of 2.8 chronic conditions per client). Following intervention, there was a statistically significant reduction in ED presentations (IRR 0.77, 95% CI 0.69–0.85), increased engagement with primary health care (IRR 1.90, 95% CI 1.78–2.03), and ambulance utilisation (IRR 1.21, 95% CI 1.07–1.38). Reductions in hospital admissions (IRR 0.93, 95% CI 0.77–1.10) and aeromedical retrievals (IRR 0.67, 95% CI 0.35–1.20) were not statistically significant. Conclusions: This study demonstrates the short-term impacts of community-led case management extending beyond the hospital setting, to address causes of recurrent ED presentations among people with complex social and medical backgrounds. Improving engagement with primary care is a particularly important outcome given the national impetus to reduce preventable hospital admissions.

## 1. Introduction

In Australia, Aboriginal and Torres Strait Islanders and people in remote areas suffer profound social and health disparities [1,2,3]. Katherine is a sparsely populated region in the tropics of Northern Australia (NT), covering 337,000 square kilometres and encompassing 19 tribal nations, and defined by some of the worst indicators of both social and health disparities anywhere in Australia. Complex forces shaped by colonisation have influenced the development of the region over the last 150 years—tribal affiliations, geography, attitudes and behaviours of early settlers, and state and federal policies. Homelessness is a longstanding problem that began with colonisation when people were displaced from ancestral land, and remains extreme in this region at 31 times the national average [4]. In the harsh tropical environment, housing insecurity leads to exposure to extreme weather and is likely to increase hospital utilization [5].

In nearly every hospital emergency department (ED) around the world, the dilemma and resource implications pertaining to frequent attenders (FAs) is common. Regardless of context, each presentation represents a request for help, a unique opportunity to engage a vulnerable person often struggling to access mainstream health services, usually with many contributing issues. The challenge for hospitals is to reframe these moments as an opportunity to restructure services to more effectively address the needs of these people.

The extreme health disparities suffered by Indigenous people testify the failures of ‘western health care’, and this is deeply evident in Katherine. One of the most difficult groups to engage in this community are FAs who disproportionately experience homelessness, alcohol misuse, and multiple comorbidities, and in this hospital represent a cohort of less than 250 people who constitute 13% of the 18,000 ED presentations per year [5]. Programs elsewhere aimed at reducing recurrent hospital use by FAs have received considerable health sector attention in Australia [6,7,8] and many other nations [9,10,11]. The key components of interventions have demonstrated the imperative of adequate case identification and management, sufficient program intensity, and integration with partner service providers [12]. Research has demonstrated the cost effectiveness of such programs in terms of reduced ED utilization [11,13] and emergency transport services [14]. There is a paucity of such research examining FA interventions in remote or tropical environments, or in communities with large proportional representation by Indigenous people.

Previous research in Katherine has defined some of the associations with frequent attendance to the hospital, and although results of this study were not statistically significant, there appeared to be a strong correlation with FA presenting even more frequently in the hot, wet monsoonal season [5]. With previous research clearly identifying Indigenous people in the remote tropical north being highly vulnerable to early impacts of climate change, it is evident that the health problems resulting from homelessness in tropical locations, particularly in rural and remote regions without adequate housing infrastructure, will worsen in the coming years [15].

**Figure 1:**
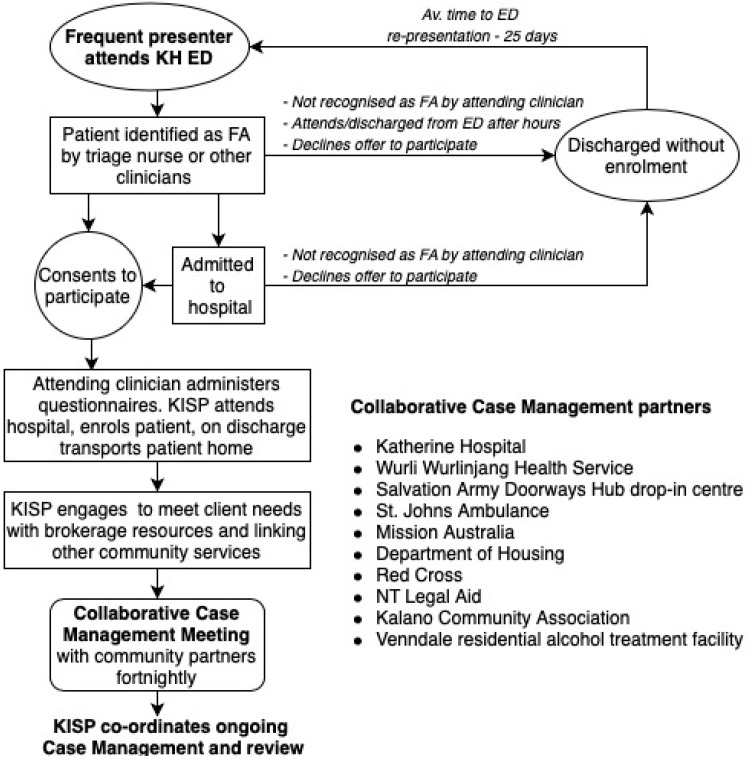
Enrolment and case management process.

The Wellness Support Pathway (WSP) was commenced in 2018 at Katherine Hospital (KH), through provision of whole-of-community, culturally appropriate case management for FAs, with threefold aims—reducing re-presentations, addressing social determinants of health, and improving health care utilisation in a community with a large Indigenous population. WSP was founded on a unique collaboration between the hospital, the town-based Aboriginal Health Service Wurli-Wurlinjang (WWHS) and an Aboriginal organisation supporting housing and alcohol rehabilitation services, named, the Kalano Community Association (KCA). WSP partners with multiple local agencies supporting the needs of socially vulnerable people (Figure 1).

This paper describes the impact of the WSP program in its first ten months and is the first study of its kind focusing on an FA intervention co-designed and implemented in a remote Aboriginal community.

## 2. Methods

### 2.1. Intervention

The intervention aimed to identify FAs to Katherine Hospital and enrol them into community-based collaborative case management to improve their well-being and redirect their access to health care to more effective services aimed at preventative health care rather than acute care. A WWHS team dedicated to case-managing participants (the Katherine Individual Support Program team, KISP) would attend the hospital and enrol participants into case management. The KISP team was designed and developed with extensive community inputs, primarily from the Aboriginal Health Service hosting this team (WWHS), with inputs from multiple Aboriginal (Katherine Region Aboriginal Health and Related Services, Sunrise Health Service, and Katherine West Health Board) and non-Aboriginal organisations (Katherine Hospital and the Northern Territory Department of Health, Drug and Alcohol Services). KISP included a team leader and three Aboriginal Liaison Officers who facilitated the provision of transport, brokerage funds covering a range of costs including food vouchers, return to country funding, camping equipment, medical and associated equipment purchases and repairs, and access to alcohol rehabilitation and crisis accommodation services.

Funding for KISP was provided via the NT Department of Health through the National Partnership Community Safety Implementation Plan [16]. Governance was through a three-tiered structure that incorporated clinical and corporate governance, with the top tier overseeing corporate governance and consisting of the four main partners in the consortium (Katherine Region Aboriginal Health and Related Services as the facilitating partner with the Department of Health, WWHS, KH, and KCA as the consortium members). The second tier consisted of organisations providing services to this vulnerable population and provided feedback and collaborative buy-in (consisting of all relevant agencies in town), and the third tier was the Collaborative Case Management Group that worked directly with the KISP clients (Table 1). The WSP and the hospital did not receive any funding.

FAs to KH were identified within the hospital by hospital staff and offered enrolment through a signed research participation consent. The first contact between KISP and participants always occurred within the hospital and included KISP transporting the participant home to make an initial assessment of the living situation.

KISP continued ongoing management and co-ordinated all the community care providers involved with each participant through a partnership with the Collaborative Case Management Group (CCMG) as described in Figure 1.

The CCMG met fortnightly and each newly enrolled participant’s case was discussed in detail, followed by other clients with active needs as determined by the CCMG members, in a structured meeting with a comprehensive confidentiality framework.

### 2.2. Study Population and Participant Enrolment

FAs were defined as six or more presentations in the previous 12 months to any NT ED. Given previous research from the Katherine Hospital, it was envisaged that there were between 250–300 frequent attenders using the hospital each year, with approximately 50–80 ED presentations each week [5]. Given the limited resources of the KISP team, hospital clinicians aimed to identify and refer between 5 and 8 participants each week. Study eligibility included age over 18 years and capacity to consent, i.e., no intellectual or other impairment including acute intoxication precluding capacity to understand and provide informed consent. As there was no mechanism to automatically identify FAs as they presented to the hospital, they needed to be identified manually and invited to participate by the attending doctor either in the ED or upon admission, as flagged by hospital electronic records. Staff underwent regular weekly sessions with the lead author to recognize how to identify FAs and enrol them when they presented to the hospital. Enrolment only occurred from Monday to Friday between 09:00 and 17:00. The project commenced in February 2018. This paper draws on data from participants enrolled in the first 10 months.

### 2.3. Study Design

The study design was a prospective cohort comparing pre- and post-intervention outcomes in the same individual, accounting for differences in time enrolled in the program, and presenting an interim analysis conducted 10 months into a planned 24-month follow-up period. The design was reviewed by clinicians and cultural representatives from KH, Menzies School of Health Research, WWHS, KCA, and other non-government organisations in Katherine.

### 2.4. Data Sources

Two patient questionnaires were designed by local clinicians and was pilot tested with a sample of 10 patients to ensure appropriate questionnaire content that validated scaling instruments were culturally and linguistically appropriate for the patient demographic, and to optimize layout. The enrolment questionnaire was distributed to the study participants at the hospital by the enrolling doctor and the Aboriginal Liaison Officer. This included a clinical questionnaire relating to medical reason for presentation, health conditions and comorbidities, medications (and compliance), drug use, and validated alcohol and nicotine dependence screening tools (AUDIT-C [17] and Fagerstrom [18]). Questionnaire completion would take approximately 20 min.

A non-clinical questionnaire collected patient information on tribal cultural heritage, community of origin, current address, housing status/homelessness, food security, education, disability and support services, and means of transport. Food security was assessed using a non-validated tool as there was no culturally or linguistically appropriate and validated measure available, and included: How many meals did you skip in the last week? Did you go hungry yesterday? How many days did you go hungry last week? Do you worry about where your next meal will come from? Do you worry that people will steal your food? Does anyone give you free meals? Food insecurity was defined as skipping meals two or more days per week, using a homeless food provider or free meals provided, being hungry yesterday, and being worried about next meal or about food theft.

Homelessness was defined as per the Australian Bureau of Statistics definition, including primary, secondary, and tertiary homelessness, by which definition a person is considered homeless if they do not have alternatives to their current living arrangement, being that they are in a dwelling that is inadequate, they have no tenure, or they do not have control of or access to space for social relations [19].

Additionally, individual linked data for each FA was collected from five organisations to enable before/after analysis of key health and social outcomes. Hospital data and mortality status were sourced from the NT Department of Health hospital database for each individual. Emergency transport data for each episode of transport was provided by St. John Ambulance (SJA) and CareFlight Aeromedical Retrieval Service (within Katherine and across the NT, respectively). Primary care data were provided by the three Aboriginal primary care services in the region (WWHS provides primary health services to the urban population around Katherine township, Katherine West Health Board services remote communities to the west, and Sunrise to the east). Each data set included the number of episodes of contact for each client 12 months prior to and the period since enrolment.

### 2.5. Statistical Analysis

Incident rate ratios were calculated to compare outcomes pre- and post-enrolment in the WSP using Stata 15.1 (Statacorp). Outcomes included the number of ED presentations, hospital admissions, and episodes of primary care.

Pre- and post-enrolment hospital data were collated by the principal researcher and cross-checked independently by a second researcher and included the number of ED presentations and admissions in the 12 months prior to and since the date of enrolment.

### 2.6. Ethics Approval

WSP is the Human Research and Ethics Committee approved research arm of KISP, an NT Department of Health-funded pilot intervention for a collaborative program to address the needs of people who were at risk of homelessness and alcohol misuse with high health needs (Menzies HREC reference 2017–2840).

Consent was obtained from individual patients capable under Australian law of providing informed consent for WSP research participation including opt-in data sharing with other agencies.

## 3. Results

### 3.1. Intervention Structure and Governance

Given the innovative nature of this collaborative program, commencement of enrolment of participants, performance protocols, and staffing of the KISP team were incremental. The KISP team developed an enrolment package for clients which included food vouchers, camping equipment, and flexible funding for urgent items of need in collaboration with the Kalano Community Association which provided a total of 579 nights of emergency accommodation over the study period.

The CCMG meetings were held fortnightly with a total of 20 meetings over the study period, with an average of 12.8 individual participants attending each meeting representing up to 12 community and government organisations. Approximately 15 cases were discussed at each meeting including all new enrolees and any other participant with active needs. The KISP team chaired and actioned items arising from each meeting and continued community-based case management between meetings.

### 3.2. Participant Demographics

Of the 109 participants, 54% were women and 91% were Indigenous, with an average age of 51 years (Table 2). At the time of analysis, ten (9%) clients had died. At the time of analysis, mean time from enrolment was 20.7 weeks with a median of 20.4 weeks, ranging from 2 to 40 weeks’ participation. There were high rates of homelessness and overcrowded housing, with a 64% composite homelessness rate. Food insecurity was high (60%) and access to transport was poor (only 20% having access to a car, in a town with no public transport). There were high rates of alcohol misuse as defined by AUDIT-C scores of 4 or more (64%), and smoking (63%). Whilst there was low amphetamine, opioid, and solvent use, cannabis use was found to be common amongst this demographic.

**Figure 2:**
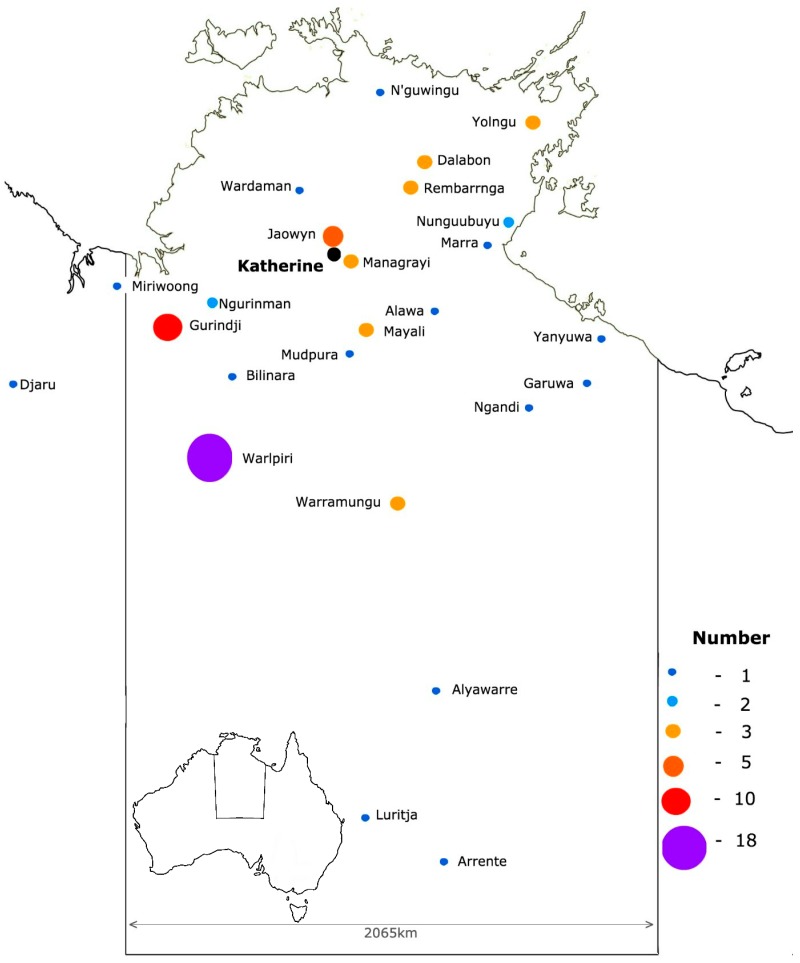
Geographic distribution of the tribal groups * of participants (* Sixty-eight participants identified as tribal origin within the NT, six participants were outside NT, 25 participants identified as Aboriginal with origin not defined, and 10 participants identified as non-Aboriginal).

The cultural demographics were complex as outlined in Figure 2. The participants comprised over 29 Indigenous tribal nations, only 15% were from communities close to the Katherine town, and 85% came from communities in more remote locations. Communities to the west of Katherine, particularly Gurindji and Warlpiri people, were over-represented.

### 3.3. Health Conditions

There was an average of 2.8 chronic health conditions per participants. Only three participants had no comorbidities and 51 (47%) had three or more comorbidities (Table 3). The highest contributor to comorbidity was drug and alcohol misuse (54%), followed by cardiovascular disease (33%), metabolic disease (30%), and chronic kidney disease (CKD, 23%), including 10% of participants being on haemodialysis. Eight of the 11 patients on dialysis met the ABS definition of homelessness and only three had concomitant alcohol misuse. There was a high rate of rheumatic heart disease (RHD, 8% participants).

Medication non-compliance, defined as not taking prescribed medications regularly, was directly related to the reason for ED presentation in 24% of the participants. Reasons for medication non-compliance included “too much tablets, forgot to take them”, “bush medicine better”, “alcohol”, “bad house”, “homeless”, and “other priorities”.

### 3.4. Changes in Service Use

Of the 109 participants enrolled, 102 had complete pre- and post- data sets regarding service use. Of these, there was a statistically significant reduction in the total number of ED representations (incidence rate ratio (IRR) 0.77, 95% Confidence Interval (CI) 0.69–0.85), from an average of 1.14 prior to 0.88 presentations per month after enrolment. There was a reduction in the number of hospital admissions, although this was not statistically significant (IRR 0.93, 95% CI 0.77–1.10) (Table 4).

In the 12 months prior to enrolment, 59% of the participants had attended the ED on more occasions than primary care, and 9% had not engaged with primary care at all. There was a significant increase in primary care engagement (IRR 1.90, 95% CI 1.78–2.03) in the period after enrolment; only 15% of the participants had attended the ED on more occasions than primary care. Before the intervention, participants used primary care services on an average of 1.79 times per month, and in the ten months after, an average of 3.84 times per month.

There was a non-statistically significant reduction in aeromedical retrievals with an IRR of 0.67 (95% CI 0.35–1.20). Conversely, there was an increase in ambulance utilisation, with an IRR of 1.21 (95% CI 1.07–1.38).

## 4. Discussion

### 4.1. Improving Health Service Utilisation

Many rural and remote towns across Australia, particularly in the tropical north, are characterised by a large proportion of Indigenous people, many of whom suffer from extreme social disadvantages, with access to health care often impaired by poor health literacy and a low sense of entitlement and expectation. The WSP has demonstrated promising interim results, including more effective utilisation of health services, secondary to the implementation of a culturally designed collaborative case management model of optimising services and supporting medical and social vulnerabilities in this population. This program reduced ED presentations by approximately 23% and increased access to primary care by 90%. Not only is this a more rational use of limited resources, but better engagement with primary care is likely to result in longer term engagement and ultimately in better health. Although this preliminary analysis has not demonstrated a reduction in emergency transport services, the trends show a reduction in utilisation in high-cost aeromedical retrievals.

It is hypothesised that the true effects of WSP will be more evident in future analyses, as the protective factors of the program slowly mitigate years of exposure to environmental, social, psychological, and medical risks.

The results of this study demonstrate an increase in ambulance utilization after program commencement. The patterns of utilization demonstrated an increase in ambulance use by those with a higher burden of comorbidity who presented less frequently, and a decrease in those with low levels of comorbidity who had alcohol use disorder and who presented more frequently. We hypothesize that the increased use in those with more comorbidity is a result of increased health literacy as a consequence of the program, and although not statistically significant, we believe the results suggest that ambulance utilization efficiency has actually improved. Similarly, although not statistically significant, given the low numbers, there was a reduction in aeromedical retrievals. Even if these numbers are low, given the high costs, any reduction in aeromedical retrieval will have significant implications for economic sustainability of the program.

### 4.2. Social Drivers of Chronic Disease

This study, which enrolled Indigenous people living in remote locations, is consistent with previous urban-based research demonstrating high rates of comorbidities in FAs [20]. The consequences of homelessness and overcrowded housing that is so prevalent in Indigenous communities across Australia, particularly in tropical environments, exacerbate the severity of illness and need for hospital care. For example, a key driver of RHD is overcrowded housing and associated high streptococcal carriage and untreated infections; rates of RHD in this study were much higher than baseline rates within the NT, even though prevalence in the remote NT is amongst the highest in the world, suggesting overcrowded housing since childhood for WSP participants [21].

Of serious concern is the fact that the majority of Indigenous people on dialysis in this study were homeless. In most regions of Australia, it is assumed that people on dialysis have ready access to food and transport, and that they live in a clean and safe home.

It is also noted that only 3 of the 11 patients on dialysis had concomitant alcohol misuse, compared to 64% of the cohort. It is hypothesised that this reflects a pattern within the entire cohort of participants, who can be grouped into two categories: roughly a third of FAs suffer serious complex health conditions with frequent exacerbations and minimal alcohol misuse, and two-thirds who are driven repeatedly to hospital by alcohol-related harm.

Given that 9% of participants had died by ten months of study commencement, it is important to recognise that in small and remote towns like Katherine servicing proportionally large Indigenous populations, the community hospital is often the first point of call for end-of-life care. For the one-third of FAs driven to repeat presentation by an advanced disease, avoiding ED is not only a more efficient use of resources, it is consistent with international research on Indigenous palliative care priorities regarding the importance of dying close to home and family [22]. Our program allows people to access care in a more planned, dignified manner.

Despite high medical comorbidities, mental illness was only reported in 15% of the participants, which most likely represents under-reporting or under-diagnosis. In a mainly indigenous population with a high-level exposure to trauma, it is hypothesised that mental health has not been given the priority it deserves, neither recognised by those who are suffering from its affliction nor by clinicians providing care.

Previous research has demonstrated mixed results regarding case management programs aimed at addressing the complex needs of frequent attenders [8,10,11,23]. The demographics of the Katherine population is starkly different to any other previous study, and it is not surprising that these results are impressive, given the opportunity to address such proverbial low hanging fruit. We also believe that a significant strength of this program compared to others reported in the literature was the implementation of targeted whole-of-community case management, tapping into pre-existing service providers who are vital to health outcomes long before hospitalization occurs. We hypothesize that hospitals servicing highly disadvantaged populations, such as the many hospitals in the north of Australia with large Indigenous populations, are well placed to tailor such collaborative case management programs in effective ways.

### 4.3. Embracing Complexity

Solutions to entrenched inequities of Indigenous disadvantage in Katherine are complex. The 29 tribal nations represented in this pilot have had different experiences shaped by the process of colonisation. These challenges are not at all homogenous; social, demographic, logistic, and cultural constructs affect solutions to providing housing and accommodation. Housing insecurity was identified as the greatest challenge to improving population health in this region.

Almost universally, ED FAs represent disadvantaged groups with complex needs who often utilise health care in ways that could be more efficiently structured. In rural and remote hospitals across Australia, Indigenous people make up a significant proportion of those in such disadvantaged groups. Australian EDs are ideally suited to provide complex discharge planning for such people, and programs like the WSP that engage with community-based agencies and provide services can be beneficial in terms of both improved health outcomes and more efficient use of resources.

In the context of a complex political, social, and cultural environment, the implementation and evaluation of the WSP itself was challenging. Given the profound health and social disparities, community resistance to ‘research’ was understandable. Logistically, undertaking research in remote communities like Katherine is difficult. The challenges included developing complex programs with limited access to academic support, recruiting and retention in remote areas, and fostering collaborative inter-organisational relationships in a town plagued with inequity.

### 4.4. Limitations

Research of this kind and in a community context such as Katherine is not without limitations. With a pre-post intervention study design and only 10 months of follow up data, longer term effects on service use cannot yet be determined. There are potential confounders that could not be controlled for, such as the establishment of the first ever homeless drop-in centre in Katherine during the intervention period. Time pressures and resource limitations within the ED meant that there were gaps in enrolment information, collected predominantly by acute care clinicians. Only two FAs invited to participate in this study declined to do so. Less than 10% of FAs who presented to Katherine Hospital each week were identified and offered enrolment into the study, either because they were not identified as an FA by the treating clinician, or because they presented after hours or on the weekend when the WSP was not enrolling participants. However, given the cyclical nature of FAs, over the study period it was estimated that approximately 50% of all FAs who had regularly attended KH were enrolled onto the program.

## 5. Conclusions

The challenges facing Aboriginal people in the Katherine region and other towns and communities in the remote tropical north of Australia are extreme. The WSP is a grass roots community-driven attempt to address the social determinant needs of a highly vulnerable demographic in the region. We found a significant reduction in ED presentations and increase in engagement with primary health care following the intervention, which represents more efficient and sustainable engagement with the health system. This intervention has demonstrated that EDs are ideally situated to go beyond band-aid solutions and address the deep social roots driving the bigger picture, implementing the right care, in the right place, at the right time, one very vulnerable person at a time.

## Figures and Tables

**Table 1:** CCMG membership.

KISP Collaborative Case Management Group
Katherine Hospital
Wurli-Wurlinjang Health Service
Kalano Community Association
St. John’s Ambulance
Mission Australia
Red Cross
Salvation Army Doorways Hub homeless drop-in centre
NT Department of Housing
Northern Territory Legal Aid
Venndale Residential Alcohol Treatment Facility
Sommerville Community Services
Catholic Care

**Table 2:** Demographics and social indicators at enrolment.

**Demographics**	
Age	51yrs (range 23–86)
Sex (*n* = 109)	54% female
Indigenous	91%
**Drug and alcohol use**	% of participants
Alcohol AUDIT-C score (*n* = 96)	5.6 (mean)
0	28%
1 to 2	4%
3 to 4	4%
Greater than 4	64%
Smoker (*n* = 93)	63%
Other drugs (*n* = 83)	19%
Cannabis	17%
Amphetamines	1%
Volatile substance (petrol fumes, other inhalant)	1%
Benzodiazepine	1%
**Medical or social vulnerability**	% of participants
Medical/social vulnerability contributing to presentation (*n* = 75)	
Medical	20%
Social	17%
Both Medical and social	63%
**Social issues**	% of participants
Food insecurity (*n* = 83)	60%
No access to transport (*n* = 80)	80%
Domestic violence (*n* = 69)	13%
Accommodation (*n* = 90)	
Meets ABS definition of homelessness	64%
Consider themselves homeless	51%
Living rough (no access to a dwelling)	26%
Lives in a house	64%
Lives in supported accommodation	2%
Lives in a temporary shelter	4%
Lives between multiple dwellings/locations	36%
Those living in a house (*n* = 64)	
Average number of people per room	2.3 (range 1–6.6)
Number with >2.5 people per bedroom	42%

**Table 3:** Medical comorbidities.

Medical Comorbidities (n = 103, Mean 2.8 per Participant, Range 0–10)	% of Participants
Drug and alcohol addiction	54%
Cardiovascular disease	33%
Metabolic disease	30%
Renal disease	23%
(on dialysis)	10%
Neurological disease	18%
Respiratory disease	18%
Infectious disease	16%
Liver/Gastrointestinal tract disease	15%
Mental health	15%
Physical disability	10%
(Rheumatic Heart)	8%
Hearing/vision impairment	8%
Malignancy	4%
Palliative care needs	4%
Autoimmune disease	2%
Pregnancy	0%
Non-compliance contributing to index presentation (*n* = 72)	51%

**Table 4:** Before-and-after impact of WSP.

Before and after Event	IRR	95% CI	Average of Episodes per Month	
			Pre-Enrolment	Post-Enrolment
ED presentations	0.77	0.69–0.85	1.14	0.88
Hospital admissions	0.93	0.77–1.10	0.35	0.35
Primary care contact episodes	1.90	1.78–2.03	1.80	3.80
Careflight retrievals	0.67	0.35–1.20	0.05	0.03
Ambulance episodes	1.21	1.07–1.38	0.62	0.72
